# Genetic architecture of adaptive radiation across two trophic levels

**DOI:** 10.1098/rspb.2022.0377

**Published:** 2022-05-11

**Authors:** Anna F. Feller, Ole Seehausen

**Affiliations:** ^1^ Division of Aquatic Ecology and Evolution, Institute of Ecology and Evolution, University of Bern, Baltzerstrasse 6, 3012 Bern, Switzerland; ^2^ Department of Fish Ecology and Evolution, Centre of Ecology, Evolution and Biogeochemistry (CEEB), Eawag Swiss Federal Institute of Aquatic Science and Technology, Seestrasse 79, 6047 Kastanienbaum, Switzerland

**Keywords:** Lake Victoria, adaptive radiation, cichlids, trophic levels, genetic architecture

## Abstract

Evolution of trophic diversity is a hallmark of adaptive radiation. Yet, transitions between carnivory and herbivory are rare in young adaptive radiations. Haplochromine cichlid fish of the African Great Lakes are exceptional in this regard. Lake Victoria was colonized by an insectivorous generalist and in less than 20 000 years, several clades of specialized herbivores evolved. Carnivorous versus herbivorous lifestyles in cichlids require many different adaptations in functional morphology, physiology and behaviour. Ecological transitions in either direction thus require many traits to change in a concerted fashion, which could be facilitated if genomic regions underlying these traits were physically linked or pleiotropic. However, linkage/pleiotropy could also constrain evolvability. To investigate components of the genetic architecture of a suite of traits that distinguish invertivores from algae scrapers, we performed quantitative trait locus (QTL) mapping using a second-generation hybrid cross. While we found indications of linkage/pleiotropy within trait complexes, QTLs for distinct traits were distributed across several unlinked genomic regions. Thus, a mixture of independently segregating variation and some pleiotropy may underpin the rapid trophic transitions. We argue that the emergence and maintenance of associations between the different genomic regions underpinning co-adapted traits that evolved and persist against some gene flow required reproductive isolation.

## 1. Introduction

The causes of the massive variation in the rates at which species diversity evolves and species-rich communities assemble in different evolutionary lineages remains one of the most fundamental questions in evolutionary biology. The East African cichlid fish adaptive radiations provide excellent systems to investigate factors contributing to such variation. The three largest lakes in this region, Lakes Victoria, Malawi and Tanganyika, each harbour several hundred species of cichlids that have evolved within the confines of these lakes (reviewed e.g. in [1,2]). In Lake Victoria alone, approximately 500 haplochromine cichlid species have evolved in less than 20 000 years [3,4]. Lake Victoria cichlids can be grouped into at least 16 different trophic groups, including detritivores, phytoplanktivores, plant eaters, molluscivores, insectivores, paedophages and piscivores, all but one of which comprise several to many species [4–6]. This staggering trophic diversity is replicated, with some modifications, across all three major radiations, and across multiple smaller radiations in smaller lakes (reviewed in [7]).

One hallmark of the East African cichlid radiations is the coexistence of many closely related species in full sympatry [7]. Any one habitat patch in the lakes is shared by dozens of species [4,5,8]. While they have retained the ability to feed opportunistically when a profitable food source is abundant, many of these species feature specialized morphologies for one specific source of food [9–11]. These hyper-diverse sympatric communities even make up entire food-webs spanning three trophic levels, including herbivory and piscivory [4–6,8]. The radiation across three trophic levels starting from insectivore ancestors [5,12] was thus key to the evolution of large species richness in these radiations [7]. Herbivory has evolved from insectivory independently in each of these radiations, and transitions between insectivory and herbivory may have occurred several times even within the youngest of the radiations [13]. In other young fish radiations, transitions between invertivory and herbivory are unusual. For instance, despite a large literature on adaptive radiation in postglacial fishes, not a single case of evolution of herbivory has been reported from lineages as diverse as stickleback, charr or sculpins [14]. Transitions to herbivory are also absent in some older freshwater radiations of other perch-like fish, such as sunfish or darters [15], and only start to become important in old continental radiations such as the characiforms or cyprinidae [16].

The evolution of herbivory requires a complex set of physiological and morphological innovations, such as a long intestine that allows breaking down and effectively processing the more resilient and low-nutrient plant tissue [17,18], and tooth morphologies and arrangements to efficiently harvest plant matter from the substrate. To understand why haplochromine cichlids repeatedly and rapidly made evolutionary transitions between invertivory and herbivory, while the same are rare or absent in other fish radiations of comparable age, we sought to understand the genetic architecture of key traits that need to change in a concerted fashion during these transitions.

Concerted (and repeated) change among several co-adapted traits could be facilitated if these traits are also correlated genetically (i.e. coded by a region containing several physically tightly linked loci or by pleiotropic loci [19,20]). For instance, supergenes (tight clusters of two or more loci each responsible for a different trait) can regulate adaptive phenotypes by linking sets of co-adapted alleles or alleles with beneficial epistatic interactions (reviewed in [20]). Once in place, such supergenes can also spread between populations and species via introgressive hybridization. However, mutations occurring in such a genomic region that affect all correlated traits, may rarely be beneficial, implying that supergenes may be constrained in their further evolution [21–23]. It is for these reasons that some have suggested that an intermediate level of integration and a modular organization may constitute the most evolvable phenotypes and genetic architectures [24], and also those most conducive to species differentiation in the absence of complete geographical isolation [25].

Quantitative trait locus (QTL) mapping studies of functional morphology in African cichlids paint a mixed picture. Some studies found that highly integrated structures within confined anatomical regions such as the lower jaw mapped to the same genomic region [26,27]. Traits previously thought to be evolutionarily decoupled such as the oral and pharyngeal jaws have also been shown to be linked genetically [28]. On the other hand, some studies mapping several functionally integrated, but anatomically separate aspects of foraging morphology [29,30], found minimal overlap of QTLs between the different aspects, suggesting modularity in the genetic architecture underlying these traits. Moreover, distinct genetic architectures have been found for the same traits in different crosses from the same lake (e.g. lower jaw lever in two different Lake Malawi crosses [26,31,32]). This could on the one hand be due to non-biological reasons such as limited power (e.g. [33]). Alternatively, it could imply that there are many loci in these systems that can underlie the same functional traits. The latter would point to a role of genotypic redundancy—when more than one genotype can produce the same phenotype or function [34]. The presence of a high level of segregating redundancy [34] in cichlids may not be surprising since the major haplochromine radiations have evolved from hybrid populations between distantly related species [35,36], and repeated bouts of admixture have also been demonstrated to have occurred within each radiation [35,37–39]. Recombination and sorting of old admixture-derived variation following the colonization of the newly formed Lake Victoria has provided high levels of segregating functional variation and—in association with many new ecological opportunities—facilitated the rapid adaptive radiation within this lake [35].

Here we use an interspecific cross between a representative of a species-rich genus of morphologically specialized herbivores (epilithic algae scrapers), and a representative of another species-rich closely related lineage of rock-dwelling invertivores, to characterize the genetic architecture associated with the transition between these trophic groups. We performed QTL mapping on a total of 22 morphological traits including linear morphological distances, several aspects of tooth morphology and dentition, and intestine length. We additionally mapped 15 male nuptial colour traits (key traits in pre-zygotic reproductive isolation in this system [40,41]).

## 2. Material and methods

### (a) Experimental cross

*Neochromis omnicaeruleus* is a representative of a lineage of morphologically specialized epilithic algae browsers. This trophic group is confined to life on rocky reefs, where they feed by dislodging firmly attached filamentous algae from rocks [42,43]. Currently, approximately 15 species are known in the widespread genus *Neochromis* [4], up to five of which can be found in sympatry on a single reef. The group as a whole is characterized by a steep dorsal head profile, a short head, closely spaced upright sub-equally to equally bicuspid outer row oral teeth that are movably implanted, three to eight rows of inner teeth in the oral jaws barely separated from the outer tooth row by any gap, and a long and coiled intestine [44,45]. Epilithic algal scrapers with similar trait combinations in the Lake Victoria radiation have additionally been described in the genera *Mbipia*, *Lithochromis* and *Paralabidochromis* [4]. Similar combinations of traits also define epilithic algal scrapers in Lakes Malawi and Tanganyika [8].

*Pundamilia* sp. ‘nyererei-like’ is a representative of a lineage of insecti-/zooplanktivores that are also restricted to rocky reefs and islands, but are characterized by a dorsal head profile that is rather shallow and straight or slightly concave, a rostrally upward inclined mouth gape, a long head, widely spaced unicuspid outer oral jaw teeth that are strongly recurved and firmly implanted, and mostly only two rows of inner teeth separated from the outer tooth row by a distinctive gap [4,45]. *Pundamilia* sp. ‘nyererei-like’ is restricted to the Mwanza Gulf in southern Lake Victoria where it likely evolved from a hybrid population of *P. pundamilia* and *P. nyererei* [46], two species that are both widespread and fully sympatric with *N. omnicaeruleus* (see electronic supplementary material, figure S1).

**Figure 1.**
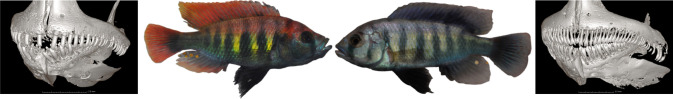
The crossed species. Photos of representative male individuals and CT scan images of the oral jaws of the two parental species used in the experimental cross: *Pundamilia* sp. ‘nyererei-like’ from Python Island (left) and *Neochromis omnicaeruleus* from Makobe Island (right). The *N. omnicaeruleus* male is the individual used as P in the cross. See electronic supplementary material, figure S1 for photos of representative female individuals and a distribution map. Photos by Oliver Selz and Ole Seehausen. CT scan images by: Mikki Law. (Online version in colour.)

These two species (figure 1; electronic supplementary material, figure S1) were crossed in the laboratory to produce second-generation hybrids (F_2_s). We used a *N. omnicaeruleus* male from our lab population bred from fishes caught at Makobe Island in Lake Victoria in 2010, and a *Pundamilia* sp. ‘nyererei-like’ female from our laboratory population bred from fishes caught at Python Island in Lake Victoria in 2003. Two first-generation hybrid (F_1_) pairs were then mated repeatedly to generate several clutches of F_2_s over a time of 3 years, resulting in a total number of approximately 213 F_2_s belonging to two full-sib families (150 males, 33 females and approx. 30 juveniles of unknown sex that had died of natural causes in the aquaria and were not used in any analyses). We currently do not know the cause of the distorted sex ratio. The rearing protocol was as described in [47]. All fish were bred and maintained in a large re-circulation aquarium system with a water temperature of 24–26°C and a 12 : 12 h light/dark cycle and fed an ad libitum diet of flake food once a day, white mosquito larvae once a week, and *Mysis* once a month. All F_2_s were reared to a minimum age of 1 year before further processing.

### (b) Colour photos and specimen processing

The protocol to obtain standardized colour followed the one in [47], and is described in detail in the electronic supplementary material, Methods. After euthanasia, we removed the right pectoral fin of every individual and stored it in 98% ethanol for later DNA extraction, and we cut open the ventral body wall to aid fixation of the guts. We fixed the specimens in 10% formalin (for min. three weeks) and then transferred them to 70% ethanol for storage.

### (c) External morphology

Using digital callipers, we measured 16 linear distances that describe the external morphology of head and body (following [48]) on the F_2_ specimens (electronic supplementary material, figure S2a). The distances were: standard length (SL), body depth (BD), head length (HL), head width (HW), lower jaw length (LJL), lower jaw width (LJW), snout length (SnL), snout width (SnW), eye length (EyL), eye depth (EyD), cheek depth (ChD), pre-orbital depth (POD), inter-orbital width (IOW), pre-orbital width (POW), caudal peduncle length (CPL) and caudal peduncle depth (CPD) (electronic supplementary material, figure S2a). We measured each trait twice, and if the deviation between the two measurements exceeded 5%, we added a third measurement. We then calculated the mean of the two (or the two closer) measurements. All 15 measured traits were significantly correlated with SL (‘cor.test’ function in R [49]), and we thus used the residuals of linear regressions (‘lm’ function in R) of each log-transformed trait against log-transformed SL in subsequent analyses. Except for CPD we did not detect any significant interaction of sex with SL (‘anova’ function in R), and so we performed size correction for males and females together.

### (d) Tooth morphology and dentition

We scored cusp shape of 10 teeth in the outer row of each oral jaw (lower and upper oral jaw), five on each side starting the count from the mid-proximal point of the jaw. Each tooth received a score between 0 (unicuspid) and 1 (sub-equally to equally bicuspid), with three steps in-between: weakly bicuspid (0.25), very unequally bicuspid (0.5) and unequally bicuspid (0.75) (see electronic supplementary material, figure S2b). We then summed up all scores in one jaw and divided this overall score by the number of scored teeth (up to 10, but excluding broken, missing, and tricuspid teeth), resulting in an overall tooth shape score of between 0 and 1 for each oral jaw. We also scored the density among these front row teeth in both jaws, assigning 0 to a large space between the outer row teeth (i.e. one extra tooth would fit in between two teeth), 1 to no space between the outer row teeth (i.e. the tooth crowns touch those of adjacent teeth) and 0.5 to an intermediate space. In the lower jaw, we additionally counted the number of inner tooth rows, and we scored the extent of the gap between the outer and first inner tooth row (0, none; 0.5, small; 1, moderate; see [45]). In total, we scored two tooth shape and four dentition traits.

### (e) Intestine length and confirmation of sex

To measure intestine length, we removed the whole alimentary canal from the F_2_ specimens, and then unrolled the intestine and laid it out on millimetre paper, taking care not to stretch the tissue. We measured the distance from the posterior end of the stomach to the anus to the nearest 0.1 mm. While dissecting out the alimentary canal, we also confirmed the sex of every individual by visual inspection of the gonads. (None had been assigned the wrong sex.) Size correction for intestine length was performed as described for external morphology.

### (f) Trait distributions and trait correlations in F_2_ and parental species males

The same set of linear distances (with the exception of CPL and CPD) had also been previously studied on wild-caught males of *Pundamilia* sp. ‘nyererei-like’ and *N. omnicaeruleus* [50]. Only males were used because the lack of species-specific nuptial coloration in females makes identification among sympatric sets of closely related species very difficult. We took the log-transformed raw data of *n* = 98 individuals of each species from this study to compare trait distributions and trait correlations within them and between them and our (*n* = 132) F_2_ males. See electronic supplementary material, Methods for details.

### (g) Colour scores

In terms of colour, males of *Pundamilia* sp. ‘nyererei-like’ are characterized by a crimson red dorsal head surface, dorsum and dorsal fin, and by yellow flanks (figure 1). The most common male morphs of *N. omnicaeruleus* are blue on the head, dorsum, flanks and dorsal fin. Some other common morphs feature some yellow or orange on some body parts [45]. The *N. omnicaeruleus* male used as P here was mostly blue, with some yellow on the flanks and hints of yellow on the operculum and nose (figure 1). We used the same colour scoring scheme as in [47], which also features *Pundamilia* sp. ‘nyererei-like’ as parental species in one of the crosses, but crossed to a closely related and sympatric blue species. In brief, we scored the presence/absence of red or yellow in 12 different sectors on the body and fins of all F_2_ males (electronic supplementary material, figure S2c). Females of both species are cryptically coloured and lack species-specific colour patterns (electronic supplementary material, figure S1). This was also true for all the F_2_ females.

### (h) Library preparation and sequence processing

For DNA extraction from fin clips, we used Qiagen's DNeasy Blood & Tissue Kit. We then produced four RAD tag sequencing libraries following [51] with some modifications, see electronic supplementary material, Methods for full details. The libraries were single end sequenced (150 bp) on four separate lanes on an Illumina HiSeq2500 machine.

For full details on sequence processing, see electronic supplementary material, Methods. In brief, we aligned demultiplexed (Stacks v. 1.40 [52]) and quality-filtered (FASTQ Quality Filter, http://hannonlab.cshl.edu/fastx_toolkit/index.html) reads to the anchored *Pundamilia nyererei* reference genome [53] using Bowtie2 v. 2.3.2 [54]. We performed base-quality recalibration using GATK's (v. 3.7) BaseRecalibrator and PrintReads modules [55] before variant and genotype calling with GATK's UnifiedGenotyper, and then identified good quality SNPs that were homozygous fixed between the two P individuals and heterozygous in the F_1_s using BCFtools (SAMtools v. 1.9 [56]).

### (i) Linkage map construction and QTL mapping

We used the resulting set of 2544 quality-filtered SNPs and 161 F_2_s to construct a linkage map JoinMap 4.0 [57], see electronic supplementary materials for full details. Linkage groups (LGs) are numbered according to the *P. nyererei* (Pun) reference and also given the corresponding *Oreochromis niloticus* (Ore) number [53].

We used R/qtl v1.46-2 [58] to perform QTL mapping. A total of 161 F_2_s of two full-sib families (i.e. from the two different F_1_ parent couples) were included in these analyses. Family 1 contained 49 males and 23 females, family 2 contained 83 males and 6 females. Since a few markers still appeared to be very close to each other we first applied the ‘jittermap’ function to jitter the marker positions in the map. We then calculated genotype probabilities using the ‘calc.genoprob’ function with a step size of 1 for the single-QTL scans and a step size of 3 for the two-QTL scans (the latter allow to test for epistatic effects), an error probability of 0.05 and the Kosambi map function. For both the single- and two-dimensional QTL scans we used the EM maximum-likelihood algorithm with the normal model, except for sex and the colour scores, for which we used the binary model. Sex and/or family were included as additive covariates for traits showing a significant effect of either of the two in an ANOVA. Genome-wide significance thresholds were estimated using *n* = 1000 permutations for each trait. For the two-QTL scans, we ran the permutations in four batches of 250 per trait on a high-performance computing cluster (each run with a different seed). Confidence intervals were estimated with the ‘bayesint’ function (within the ‘summary’ function in the ‘tabByChr’ format). We calculated percentage of variance explained (PVE) following [59] using the formula PVE = 1 – 10^((−2/*n*)*LOD)^ × 100, where *n* is the number of individuals. We also implemented a multiple QTL approach, which did not reveal any additional insights since the resulting QTL models consisted of 1–2 loci per trait, as already detected in the single-QTL scans, with no apparent interactions (see electronic supplementary material, Methods and table S3).

## 3. Results

### (a) Patterns of trait correlations are overall similar

In all trait correlation matrices, we found a rather large number of significant trait correlations (electronic supplementary material, figure S3). The correlation matrices of each of the parental species on its own, and that of the F_2_s were all highly similar to each other. As expected, the number of significant correlations was largest and the highest correlation coefficients were observed in the matrix that combines the parental species (electronic supplementary material, figure S3c). The number of significant correlations was smallest in the F_2_s (electronic supplementary material, figure S3b), as might be expected under the experimental design. Many of the correlations expected from the combined parental species matrix (electronic supplementary material, figure S3c) were indeed present in the hybrids (electronic supplementary material, figure S3d). Some of the strongest correlations in the F_2_s were among different aspects of tooth morphology and dentition (electronic supplementary material, figure S3a).

### (b) QTLs are distributed across multiple linkage groups

**Figure 2.**
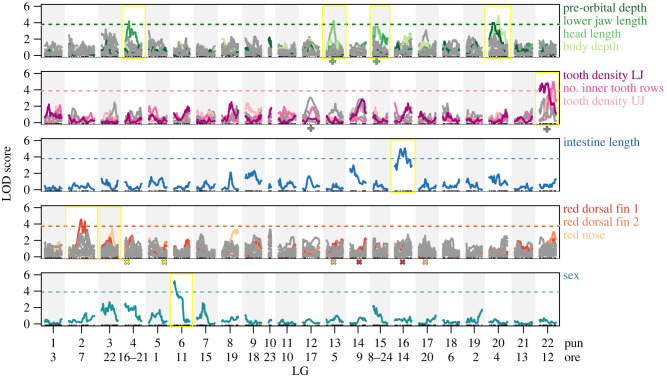
The QTLs for a suite of traits differentiating an insectivore from a specialised algae scraper are distributed across several linkage groups (LGs). Each panel shows one trait complex: (*a*) external morphology (15 size-corrected linear distances), (*b*) tooth morphology and dentition, (*c*) intestine length, (*d*) male nuptial colour (presence/absence scores of red and yellow on 12 body sectors) and (*e*) sex. Within each panel, traits with at least one marginally significant (*p* < 0.1) QTL are plotted in colour (see electronic supplementary material, table S1), traits with no detectable QTL in grey. Dashed lines represent a genome-wide significance threshold of *p* < 0.05, coloured by trait. LGs with a (marginally) significant QTL are additionally highlighted with a yellow frame. ‘Pun’ numbers are given for each LG and correspond to the chromosome numbers of the anchored *P. nyererei* reference genome, ‘ore’ numbers are given below and correspond to the *O. niloticus* chromosome numbers [53]. The plus signs below the panels indicate the approximate position of additive QTLs detected in two-QTL scans, the crosses the approximate positions of putatively interacting QTLs detected in two-QTL scans (see electronic supplementary material, table S2). (Online version in colour.)

We found four significant and one marginally significant QTLs for four of the 15 measured external morphology traits, mapping to four different chromosomes (figure 2; electronic supplementary material, table S1): one QTL for LJL on Pun-LG4/Ore-LG16-21 (LOD = 4.18, *p* = 0.019), one QTL for HL on Pun-LG13/Ore-LG5 (LOD = 4.19, *p* = 0.028), an additional marginally significant QTL for HL on Pun-LG15/Ore-LG8-24 (LOD = 3.86, *p* = 0.057), one QTL for BD on Pun-LG20/Ore-LG4 (LOD = 4.81, *p* = 0.002), and one QTL for POD also on Pun-LG20/Ore-LG4 (LOD = 3.99, *p* = 0.033). The peaks for the two latter QTLs mapped to a different position on Pun-LG20/Ore-LG4 but with overlapping confidence intervals (figure 2; electronic supplementary material, table S1).

A significant QTL for intestine length was found on Pun-LG16/Ore-LG14 (LOD = 5.04, *p* = 0.006). Three aspects of dentition mapped to the same chromosome (Pun-LG22/Ore-LG12) with partially overlapping confidence intervals: tooth density in both jaws and the number of tooth rows in the lower jaw (LOD > 4, *p* < 0.05 for all three; figure 2; electronic supplementary material, table S1). While not significant, the highest peaks for tooth shape in both jaws were also seen on this chromosome.

Two-QTL scans for all these morphological traits revealed only one additional QTL pair: two additive QTLs for tooth shape in the lower jaw were detected, one on Pun-LG22/Ore-LG12 and one on Pun-LG12/Ore-LG17 (electronic supplementary material, table S2). The two QTLs for HL were confirmed in this analysis and shown to be purely additive.

We found QTLs for three of the scored colour traits (figure 2 and electronic supplementary material, table S1): two fully overlapping significant QTLs for red on the two sectors on the dorsal fin on Pun-LG2/Ore-LG7 (both LOD = 4.55, *p* < 0.05), and one marginally significant QTL for red on the nose on Pun-LG3/Ore-LG22 (LOD = 3.52, *p* = 0.088). Two-QTL scans revealed some additional and putatively interacting QTLs for red on the first sector of the dorsal fin (Pun-LG14/Ore-LG9 and Pun-LG16/Ore-LG14), for red on the nose (Pun-LGs13/Ore-LG5 and Pun-LG17/Ore-LG20) and for yellow on the cheek (Pun-LG4/Ore-LG16-21 and Pun-LG5/Ore-LG1) (electronic supplementary material, table S2).

Finally, sex mapped to Pun-LG6/Ore-LG11 (LOD = 5.2, *p* < 0.001), an LG that has not been reported to carry a sex-determining region in East African cichlids before [60] (and references therein). To identify additional sex-determining loci and the exact mode of sex determination in the two crossed species requires further work.

Percentage variance explained (PVE) of QTLs for all morphological and colour traits was between 10.6% and 14.7% (electronic supplementary material, table S1), i.e. they were all of moderate to relatively large effect. However, these effects are likely overestimated due to our modest sample sizes, consistent with the Beavis effect [61]. Given our linkage map and sample size, our statistical power to detect a significant QTL with an effect size of 10% PVE was approximately 64%. Note though that this limitation introduced a conservative bias with regard to our interpretations.

### (c) QTL effects and dominance

**Figure 3.**
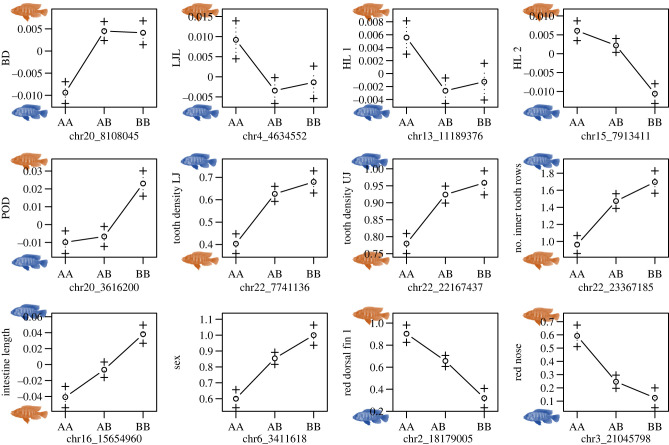
QTL effects are concordant. Shown are effect plots for all traits with (marginally) significant QTLs (electronic supplementary material, table S1; and see electronic supplementary material, figure S5 for trait distributions by genotype among the F_2_s). The AA genotype corresponds to *Pundamilia* sp. ‘nyererei-like’, the BB genotype to *N. omnicaeruleus*, AB are heterozygotes. Chr numbers correspond to Pun-LG numbers; see figure 2. If the QTL peak was on an interpolated marker, the nearest flanking marker is shown (see electronic supplementary material, table S1). The fish cartoons indicate which parental species is associated with the larger values for a given trait (red = *Pundamilia* sp. ‘nyererei-like’, blue = *N. omnicaeruleus*; see also electronic supplementary material, figure S4). Only the QTL for BD and POD are discordant. (Online version in colour.)

Most QTLs were in the expected direction. That is, in traits where wild-caught *Pundamilia* sp. ‘nyererei-like’ males have larger trait values than *N. omnicaeruleus* males, the largest trait values in the F_2_s were associated with the *Pundamilia* sp. ‘nyererei-like’ (AA) genotype, and the smallest with the *N. omnicaeruleus* (BB) genotype, and vice versa depending on the trait (figure 3; electronic supplementary material, figures S4 and S5). Only the QTLs for BD and POD did not conform to this pattern. In POD however, the difference in means between the wild-caught individuals of the two species was small, and this was also one of the few traits in which transgressive trait values were observed in the F_2_s. We broadly define transgressive trait values as lying outside the combined parental species' distribution (see electronic supplementary material, figure S4). Note however that transgression could also be caused by comparing wild-caught to laboratory-raised individuals. Most QTLs showed signs of dominance (or even slight overdominance) except for the QTLs for intestine length and red on the dorsal fin (figure 3). Most dominance effects were attributed to the *N. omnicaeruleus* alleles.

## 4. Discussion

We asked if an explosive trophic radiation that involved repeated transitions between trophic levels might have been facilitated by genetic architectures involving physical linkage or pleiotropy among co-adapted trophic traits. While we found indications of physical linkage or pleiotropy within trait complexes, the QTLs for distinct functional traits that distinguish specialized herbivores (epilithic algae browsers) from invertivores were distributed across several chromosomes.

With our sample sizes of 127–161 F_2_s, we had insufficient power to detect QTLs of small effect and limited power to detect QTLs of moderate effect, which is reflected in the absence of QTLs for more than half of the mapped morphological traits. To obtain much larger sample sizes in interspecific crosses of mouthbrooding haplochromine cichlids is challenging, however. In our case, it took over three years to obtain around 200 F_2_ individuals, of which not all survived to adulthood (about one year of age). A limited sample size can bias QTL detection towards larger effect variants, and lead to the overestimation of QTL effects [61]. In our study, we cannot make any statements about the locations or effects of undetected smaller effect loci. Our main finding still holds though: the QTLs with the largest and detectable effects for distinct traits (i.e. linear morphological traits versus tooth/dental morphology versus intestine length) mapped to physically unlinked regions in the genome. Recent QTL mapping studies of functional morphology in haplochromine cichlid hybrids between species from within the same trophic level reported similar findings. For instance, a large number of small-effect (mostly additive) loci were found to underpin both lip and head morphology, with little overlap, in a cross of the two Lake Victoria invertivores *Paralabidochromis chilotes* and *Pundamilia nyererei* [30]. Similarly, [29] found several QTLs each for three types of traits (craniofacial, fin and body shape) with little overlap in a cross of the two epilithic algae eating Lake Malawi haplochromines *Labeotropheus fuelleborni* and *Tropheops* sp. ‘red cheek’.

Here however, we also found indications of physical linkage or pleiotropy, possibly among some of the linear traits (BD and POD) and especially among different aspects of oral jaw dentition and tooth morphology. The QTLs with the largest effects for tooth density in both oral jaws and the number of inner tooth rows in the lower jaw were located on the same linkage group (Pun-LG22/Ore-LG12) with partially overlapping confidence intervals. The highest (albeit non-significant) peaks for tooth shape in both jaws were also located on this LG. This linkage group has not previously been reported to be associated with oral jaw dentition or tooth morphology in cichlids [32,62–64]. Further indications for the presence of pleiotropic effects or physical linkage might be given by patterns of trait correlations in the F_2_s, where some of the strongest correlations were among different aspects of tooth and dental morphology.

To our knowledge, ours is the first study genetically mapping intestine length in cichlids. In addition to several morphological traits in the head, differences in both tooth morphology and intestine length are strongly correlated with carnivorous versus herbivorous diets in cichlids (reviewed in [65]) and constitute important diagnostic differences between species feeding at these two trophic levels, including the two parental species of our cross [6,44]. A long intestine is a major indicator of adaptation to herbivory not only in fishes (e.g. [18,66]) but also in birds [67], mammals [68] and reptiles [69]. While expensive to maintain, having a longer gut probably serves to better take up nutrients from food that is more resilient to digestion by increasing retention time [17]. It has been shown to have a strong plastic component (e.g. [18]), which has been suggested to provide a mechanism that could facilitate trophic shifts in evolutionary radiations [18]. In our study, the QTL for intestine length is among the ones of largest effect, clearly indicating a genetic basis for this trait. We cannot rule out an additional plastic component however, as intestine length in the F_2_ males only exceeded two times their body length (SL) in two individuals (and it was shorter than two times their standard length in all others), while it has been described as up to three or four times SL in wild *N. omnicaeruleus* males [4]. These generally shorter intestines in the F_2_s may well reflect a plastic response to the common-environment aquarium food, which is made up of both plant and animal components. However, the detection of a QTL in the F_2_ and the direction of the allelic effects are clear evidence for a genetic basis of differences in intestine length between the invertivorous and the herbivorous species.

Transitioning from an invertivorous to a herbivorous lifestyle has likely happened several times in the rapid radiation of the Lake Victoria cichlids, and possibly the reverse has happened too [13]. Given that such transitions require changes in many different traits, we hypothesized that such concerted changes could have been facilitated by a ‘simple’ genetic architecture involving pleiotropy and/or physical linkage (e.g. [19,20]), especially since much of the adaptive radiation will have happened in the face of gene flow [70,71]. We found indications for some linkage/pleiotropy within trait complexes, but also that QTLs for distinct traits whose co-adaptation is functionally required to make a ‘good’ invertivore or herbivore, such as intestine length, head length, jaw length and tooth morphology, mapped to physically unlinked regions of the genome. Thus, a mixture of pleiotropic effects and independently segregating variation containing at least some moderate effect loci, may have facilitated the rapid and repeated transitions between the two tropic levels. The required high levels of segregating functionally relevant variation containing moderate to large effect loci may have be derived from old admixture variation [35,72]. Another factor that may have facilitated transitions is the direction of dominance. For most morphological traits for which we found QTLs, the dominant allele came from *N. omnicaeruleus*, that is, the derived trophic state.

We argue that at the stage where herbivores evolved from the invertivorous ancestors, while pleiotropic effects may have favoured divergence to some degree, multi-locus LD would additionally have had to build between the physically unlinked regions in the genome that underpin distinct traits whose co-functionality is required to make a ‘good’ herbivore or insectivore. The evolution and maintenance of such genome-wide LD between multiple unlinked genomic regions in the face of gene flow would not only have required divergent selection but also reproductive isolation [73–75]. Hence, rapid speciation clearly was a prerequisite for adaptive radiation in Lake Victoria. Two observations in our study might provide evidence for divergent selection that would have needed to act during the transition from the invertivorous ancestral to the herbivorous derived state. First, the concordant direction of effects of most of the detected QTLs (figure 3) in our study are consistent with the sorting of alleles under divergent ecological selection between the two niches [76]. Second, compared to crosses between ecologically similar species [12,77] we observed little transgressive variation in our cross, which is also consistent with theoretical expectations of the sorting of alleles under divergent selection [78]. As for reproductive isolation, our species belong to lineages that are behaviourally reproductively isolated in complete sympatry [79]. Reproductive isolation among many sympatric Lake Victoria cichlids is to a large degree due to behavioural female mate choice based on male nuptial colour patterns [79–81]. Theory predicts that the evolution and maintenance of reproductive isolation in a sympatric setting should be facilitated if mate choice traits are linked with traits under disruptive ecological selection [82–84]. But interestingly, we see no evidence of physical linkage between mating traits (male colour) and functionally relevant morphological traits in our cross.

Follow-up investigations should narrow down the genomic regions identified by QTL mapping to screen for the causative variants underlying a given trait. We predict that indels—disrupting old and establishing new interactions between protein-coding genes and regulatory elements—might be involved since many of the old haplotypes in the Lake Victoria radiation have been shown to carry indels associated with specific ecologies [13].

In conclusion, we have shown that several unlinked genomic regions as well as some pleiotropy contribute to the functional morphological differences between representatives of a carnivorous and a herbivorous lineage within the rapid radiation of Lake Victoria cichlid fish. Repeated and rapid transitions between these two feeding modes and trophic levels, which were key in generating the exceptional species richness during this adaptive radiation, thus probably required the early rapid evolution of robust reproductive isolation combined with a mixed genetic architecture containing many independently segregating genetic variants, some of which with at least moderate effects on feeding ecology, and also some pleiotropy.

## Data Availability

Raw read (fastq) files for all genotyped individuals are accessible on the Sequence Read Archive (SRA) under PRJNA763171. Phenotype–genotype tables and code are available from the Dryad Digital Repository: https://doi.org/10.5061/dryad.931zcrjm2 [85]. Additional information is provided in electronic supplementary material [86].
